# Apoptosis-Related Gene Expression Profiles of Mouse ESCs and maGSCs: Role of Fgf4 and Mnda in Pluripotent Cell Responses to Genotoxicity

**DOI:** 10.1371/journal.pone.0048869

**Published:** 2012-11-07

**Authors:** Tatjana Khromov, Ralf Dressel, Iliana Siamishi, Jessica Nolte, Lennart Opitz, Wolfgang Engel, D. V. Krishna Pantakani

**Affiliations:** 1 Institute of Human Genetics, University of Goettingen, Goettingen, Germany; 2 Department of Cellular and Molecular Immunology, University of Goettingen, Goettingen, Germany; 3 DNA Microarray Facility, University of Goettingen, Goettingen, Germany; Baylor College of Medicine, United States of America

## Abstract

Stem cells in the developing embryo proliferate and differentiate while maintaining genomic integrity, failure of which may lead to accumulation of mutations and subsequent damage to the embryo. Embryonic stem cells (ESCs), the *in vitro* counterpart of embryo stem cells are highly sensitive to genotoxic stress. Defective ESCs undergo either efficient DNA damage repair or apoptosis, thus maintaining genomic integrity. However, the genotoxicity- and apoptosis-related processes in germ-line derived pluripotent cells, multipotent adult germ-line stem cells (maGSCs), are currently unknown. Here, we analyzed the expression of apoptosis-related genes using OligoGEArray in undifferentiated maGSCs and ESCs and identified a similar set of genes expressed in both cell types. We detected the expression of intrinsic, but not extrinsic, apoptotic pathway genes in both cell types. Further, we found that apoptosis-related gene expression patterns of differentiated ESCs and maGSCs are identical to each other. Comparative analysis revealed that several pro- and anti-apoptotic genes are expressed specifically in pluripotent cells, but markedly downregulated in the differentiated counterparts of these cells. Activation of the intrinsic apoptotic pathway cause approximately ∼35% of both ESCs and maGSCs to adopt an early-apoptotic phenotype. Moreover, we performed transcriptome studies using early-apoptotic cells to identify novel pluripotency- and apoptosis-related genes. From these transcriptome studies, we selected *Fgf4* (Fibroblast growth factor 4) and *Mnda* (Myeloid cell nuclear differentiating antigen), which are highly downregulated in early-apoptotic cells, as novel candidates and analyzed their roles in apoptosis and genotoxicity responses in ESCs. Collectively, our results show the existence of common molecular mechanisms for maintaining the pristine stem cell pool of both ESCs and maGSCs.

## Introduction

Embryonic stem cells (ESCs) derived from mouse pre-implantation blastocysts are pluripotent [1], [2] and have the ability to differentiate into all three germ layers [3], [4], [5]. During embryogenesis, stem cells proliferate and differentiate, while maintaining the genomic integrity to avoid the accumulation of mutations, which may subsequently damage the embryo. In line with this view, it has been proposed that ESCs might have evolved with mechanisms to protect against genotoxic stress by employing either very efficient DNA repair machinery or by inducing apoptosis when even low levels of DNA damage are encountered [6], [7], [8]. Moreover, mutation frequencies and mitotic recombination events in ESCs were shown to be 100-fold lower than in somatic cells, thus supporting the existence of efficient mechanisms against genotoxicity [9], [10].

ESCs are highly proliferative and display a distinct, short cell cycle (10–12 h) with a very brief G1 phase [6], [11]. Unlike somatic cells, ESCs were shown to contain DNA strand breaks (DSBs) marked by γH2A.X but do not activate the DNA repair system [11], suggesting their tolerance to DSBs. Moreover, ESCs do not undergo G1 arrest upon DNA damage partly due to the inactivation of p53 and low levels of cyclin dependent kinase (CDK) inhibitor p21/Waf1 [6]. Consistent with these data, it has been reported that in ESCs the checkpoint kinase, Chk2, does not phosphorylate its substrates, such as p53 and Cdc25A, leading to the lack of G1 arrest [12]. In contrast to the expected general function of p53 in DNA damage response, it has been reported that p53 mediates the repression of pluripotency gene *Nanog* upon DNA damage [13]. Repression of this core pluripotency factor therefore allows the differentiation of damaged cells and subsequent elimination through p53-mediated mechanisms [13].

**Figure 1:**
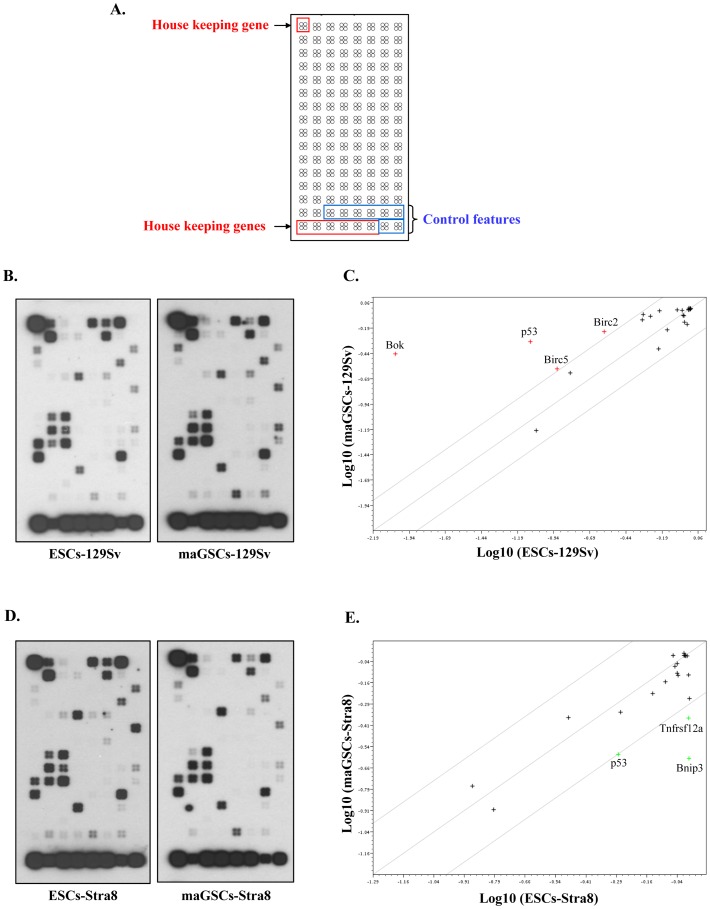
Comparative analysis of apoptosis-related genes expression profiles in undifferentiated ESCs and maGSCs. (**A**) The format of the Mouse Apoptosis OligoGEArray spotted with oligos against 112 genes known to be involved in apoptosis-related processes. Additionally, oligos spotted against housekeeping genes (red) and blank or plasmid controls (blue) are also shown. (**B**) OligoGEArray blot showing the expression profile of apoptosis-related genes in undifferentiated ESCs and maGSCs of the 129Sv genetic background. (**C**) Scatterplot analysis of genes expressed in undifferentiated ESCs and maGSCs of the 129Sv background. Expression of pro-apoptotic gene *Bok* and three anti-apoptotic genes (*p53*, *Birc2*, and *Birc5*) were upregulated in maGSCs relative to ESCs, but the expression of most genes did not substantially differ between the cell types. (**D**) OligoGEArray blot showing the expression profile of apoptosis-related genes in undifferentiated ESCs and maGSCs of the Stra8-EGFP transgenic background. (**E**) Scatterplot analysis of genes expressed in undifferentiated ESCs and maGSCs of the Stra8-EGFP background. Two pro-apoptotic genes, *Bnip3* and *Tnfsrf12a*, were slightly downregulated in maGSCs relative to ESCs, but most genes did not substantially differ between the cell types.

Multipotent adult germline stem cells (maGSCs) derived from spermatogonial stem cells (SSCs) of adult mouse testis are another example of pluripotent stem cells [14]. These maGSCs are able to differentiate into all the germ layers *in vitro* and can contribute to chimeras with germ-line transmission. Previously, to further investigate the pluripotency-related properties of maGSCs, we have examined the microRNA expression, global gene expression and proteomics analysis and have found similarities with ESCs [15], [16], [17]. Furthermore, analysis of the epigenetic features of maGSCs by global and gene-specific DNA methylation and histone modification profiling also demonstrated the similarity of both pluripotent cell types [18], [19]. The successful generation of human maGSCs might provide an alternative to ESCs in regenerative medicine applications, as the use of maGSCs can bypass both ethical and immunological issues.

In the present study, we analyzed apoptosis-related gene expression in maGSCs and found the expression pattern to be comparable to that of ESCs. We found the expression of intrinsic, but not extrinsic, apoptotic pathway genes in both cell types. We identified the exclusive expression of several pro- and anti-apoptotic genes in both pluripotent cell types, but not in their differentiated counterparts. Activation of the intrinsic apoptotic pathway cause approximately ∼35% of both ESCs and maGSCs to adopt an early-apoptotic phenotype, demonstrating a strong similarity between these cell types. We performed gene expression profiling on early-apoptotic ESCs and maGSCs and identified two candidate genes, namely *Fibroblast growth factor 4* (*Fgf4*) and *Myeloid cell nuclear differentiating antigen* (*Mnda*), which are highly downregulated upon induction of apoptosis. Further experiments revealed that *Fgf4*-knock-out (*Fgf4*-KO) cells are partially protected against induced genotoxicity.

**Figure 2:**
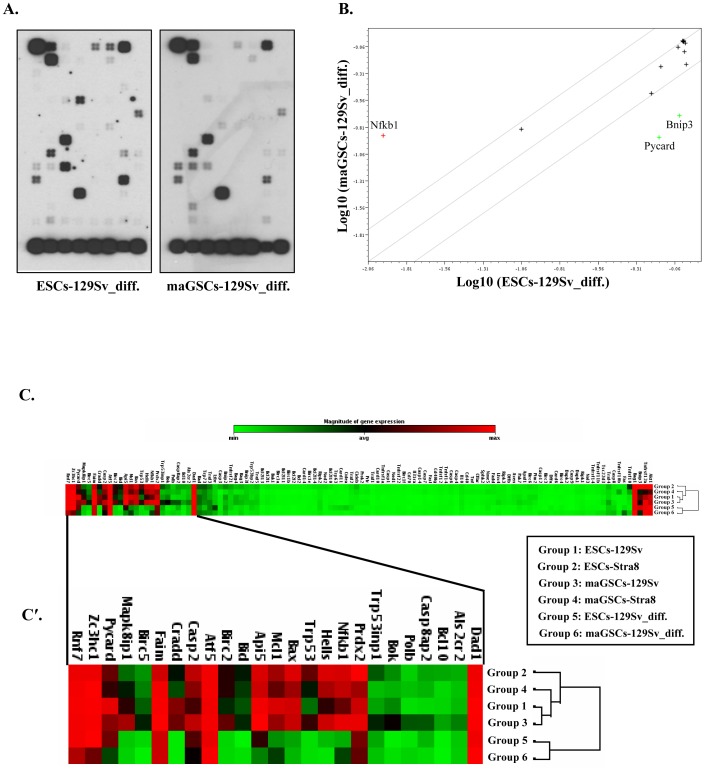
Expression profiling of apoptosis-related genes in differentiated ESCs and maGSCs. (**A**) OligoGEArray blot showing the expression pattern of apoptosis-related genes in ESCs and maGSCs of the 129Sv background that had been differentiated for 21 days with retinoic acid. (**B**) Scatterplot analysis of differentiated ESCs and maGSCs revealing similar gene expression patterns in both differentiated cell types with upregulation of *Nfkb1* and downregulation of *Pycard* and *Bnip3* in differentiated maGSCs. (**C**) Heatmap analysis of undifferentiated ESCs and maGSCs (from both the 129Sv and the Stra8-EGFP backgrounds) as well as differentiated ESCs and maGSCs, revealing all undifferentiated cell types in one cluster, while differentiated cell types are distant and clustered together. (**C′**) Specific and strong expression of several anti-apoptotic and pro-apoptotic genes in undifferentiated ESCs and maGSCs relative to differentiated cells was highlighted.

**Figure 3:**
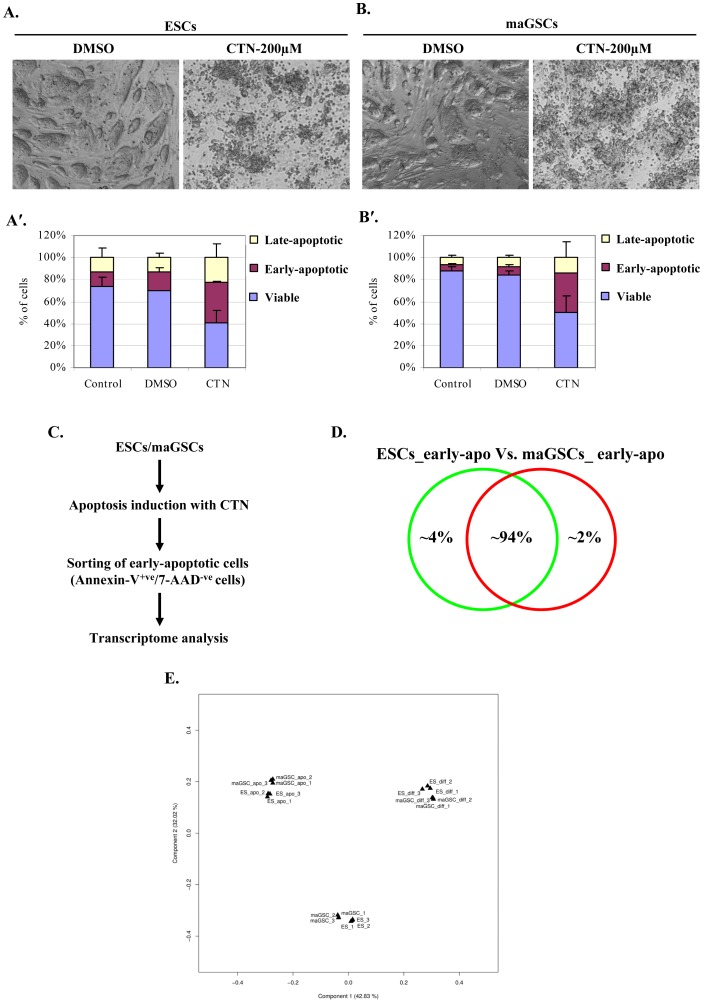
Induction of apoptosis and transcriptome analysis of early-apoptotic ESCs and maGSCs. ESCs (**A**) and maGSCs (**B**) treated with DMSO for 24 h showed typical colony morphology, whereas cells treated with CTN for 24 h lost their typical colony morphology and appeared as blebs. (**A′**) Stacked bar graph showing the flow cytometric data of annexin-V and 7-AAD staining on control (non-treated), DMSO-treated, and CTN-treated ESCs. (**B′**) Stacked bar graph showing the flow cytometric data of annexin-V and 7-AAD staining on control (non-treated), DMSO-treated, and CTN-treated maGSCs. (**C**) Outline of the strategy to identify pluripotency- as well as apoptosis-related genes in pluripotent cells. ESCs and maGSCs were treated with CTN, and early apoptotic cells (annexin-V^+ve^/7-AAD^−ve^ cells) were collected by flow cytometry. The sorted early-apoptotic cells were then used for transcriptome analysis. (**D**) The transcriptomes of early-apoptotic ESCs and maGSCs were ∼94% identical during the apoptotic response, whereas 6% genes were differentially expressed. (**E**) Principle component analysis showing the clustering of early apoptotic ESCs and maGSCs transcriptomes, which are distant to previously generated transcriptomes of undifferentiated as well as differentiated ESCs and maGSCs.

## Materials and Methods

### Generation of Constructs

The mouse Mnda expression construct, pUNO1-mMnda (Invivogen), which expresses Mnda under the control of hEF1α promoter, was linearized with *ApaL* I and then transfected into ESCs by electroporation. The shRNA expression constructs against mouse *Mnda* (Qiagen) were linearized with *Sca* I and used for transfection. To generate the *Fgf4* overexpression construct with an HA epitope tag (phEF1α-*Fgf4*_HA), the ORF of *Fgf4* was PCR-amplified from pBluescript-m*Fgf4* (Addgene) using primer pair 5′-GAATTCTGATG GCGAAACGCGGGCCG-3′ and 5′-GCGGCCGCTCAAGCGTAATCTGGAACATCGTA TGGGTAACCACCCAGTCTAGGAAGGAAGTG-3′ and cloned into the phEF1α expression vector, using the *EcoR* I and *Not* I restriction sites.

### Cell Culture

The derivation of the mouse maGSC and the respective ESC lines from the 129Sv and Stra8-EGFP backgrounds were described previously [19]. The *Fgf4*-KO ESC line was a kind gift from Prof. Rizzino [20]. Mouse embryonic fibroblasts (MEFs) were grown in standard fibroblast culture conditions. To generate *Mnda* and *Fgf4* overexpression clones (*Mnda*-OE and *Fgf4*-OE, respectively), the constructs were electroporated into ESCs and the cells were selected for neomycin resistance. To generate Mnda downregulation clones (*Mnda*-DN), the shRNA expression constructs against *Mnda* were electroporated into ESCs and the cells were selected for neomycin resistance. For differentiation experiments, the cells were cultured in medium containing retinoic acid for the indicated time points without leukemia inhibitory factor (LIF) and feeder cells.

**Table 1:** List of GO terms associated with upregulated genes in early-apoptotic cells.

S. No	GO term	Gene count	%	p-value
**1**	Sensory perceptioin of chemical stimulus	150	12	1.20E-25
**2**	G-preotein coupled receptor protein signaling pathway	184	14.7	4.60E-19
**3**	Cell surface receptor linked signal transduction	201	16.1	1.20E-11
**4**	Cell cycle process	38	3	3.10E-04
**5**	Response to DNA damage stimulus	30	2.4	4.40E-04
**6**	DNA repair	25	2	5.10E-04
**7**	Transcription, DNA dependent	15	1.2	4.20E-03
**8**	Sexual reproduction	33	2.6	5.80E-03
**9**	Chromatin organization	28	2.2	7.00E-03
**10**	Cellular response to stress	32	2.6	5.80E-02

### Oligonucleotide Microarray for Detection of Apoptosis-specific Gene Expression

Expression of apoptosis-specific genes in undifferentiated or differentiated maGSCs and ESCs was examined using Mouse Apoptosis OligoGEArray following the manufacturer’s protocol (SABiosciences). Briefly, total RNA extracted from maGSCs and ESCs was used to prepare cDNA followed by biotin 16-dUTP labelling using a GEArray True Amp Labelling kit (SABiosciences). The Mouse Apoptosis OligoGEArray membranes were then hybridized overnight with biotin-labelled cDNA. Signals were detected using a CDP-Star chemiluminescence kit (SABiosciences). Data were analyzed using the GEArray Expression Analysis Suite 2.0 (SABiosciences). For fold differences, an arbitrary threshold of 2 was chosen to signify a substantial fold change in either direction. All experiments were done with two independent biological samples.

### Apoptosis Induction, Annexin-V Staining, and Flow Cytometry

To induce apoptosis through the mitochondrial pathway, maGSCs and ESCs were cultured in medium containing citrinin (CTN) (Sigma) or DMSO (Sigma) (as a control) for either 12 or 24 h. To verify and quantify CTN-induced apoptosis in maGSCs and ESCs, annexin-V and 7-amino-actinomycin D (7-AAD) staining was performed using an Annexin V-PE Apoptosis Detection Kit I (BD Biosciences). After staining, flow cytometric measurements were performed on a FACSCalibur flow cytometer (BD Biosciences) and analyzed with CellQuestPro software (BD Biosciences). Early-apoptotic cells were sorted using a FACSCanto II (BD Biosciences). All experiments were done in three or more independent biological replicates.

**Table 2:** List of GO terms associated with downregulated genes in early-apoptotic cells.

S. No	GO term	Genecount	%	p-value
**1**	Regulation of cell proliferation	75	4.8	1.50E-05
**2**	Cell adhesion	77	4.9	2.10E-05
**3**	Oxidation reduction	85	5.4	1.40E-04
**4**	Cell death	66	4.2	4.00E-04
**5**	Phoshphate metabolic process	92	5.8	1.50E-02
**6**	Regulation of transcription	212	13.4	2.80E-02
**7**	Cellular macromolecule catabolic process	65	4.1	3.70E-02
**8**	Establishment of protein localization	69	4.4	4.20E-02
**9**	Intracellular signaling cascade	91	5.8	6.60E-02
**10**	Regulation of programmed cell death	58	3.7	7.60E-02

**Figure 4:**
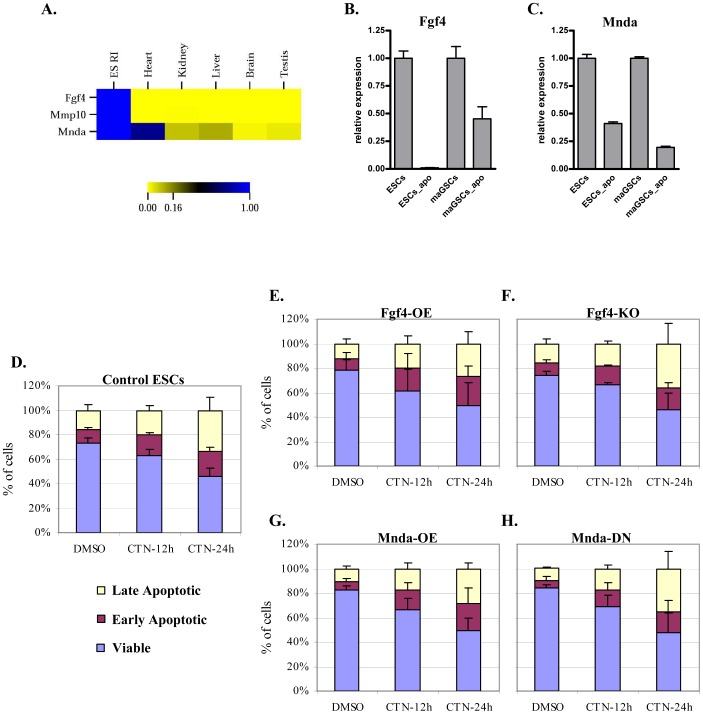
Identification and characterization of novel pluripotency- and apoptosis-related genes. (**A**) Heatmap representation of qPCR data of selected candidate genes in ESCs (ES RI) and adult mouse tissues. (**B**) The qPCR analysis confirming the downregulation of Fgf4 in early apoptotic ESCs and maGSCs. (**C**) The qPCR analysis confirming the downregulation of Mnda in early apoptotic ESCs and maGSCs. (**D–H**) Stacked bar graphs showing the percentage of viable, early-apoptotic, and late-apoptotic cells in either DMSO- or CTN-treated cells. Induction of apoptosis and analysis by annexin-V/7-AAD staining after 12 h and 24 h of CTN treatment in wild-type ESCs (**D**), *Fgf4*-OE (*Fgf4*-overexpressing) cells (**E**), *Fgf4*-KO (*Fgf4*-knock-out) cells (**F**), *Mnda*-OE (*Mnda*-overexpressing) cells (**G**), *Mnda*-DN (*Mnda*-downregulated) cells (**H**). The flow cytometry data of three or more independent biological replicates were calculated and represented as a mean ±SD. The data was analyzed for statistical significance and found no significant differences in **D–H**.

### DNA Damage Induction and Cell Cycle Analysis

For induction of DNA damage, the cells were treated with 50 ng/ml neocarzinostatin (NCS) diluted in PBS. After 30 min of treatment, the cells were washed with PBS and supplemented with culture medium. For cell cycle analysis, the cells were trypsinized after indicated time points and were washed with PBS followed by ethanol fixation at −20°C for a minimum of 2 h. After fixation, the cells were washed with PBS, resuspended in PBS containing 10 µg/ml propidium iodide (PI) and 1 mg/ml RNase A, and incubated at 37°C for 30 min. After the incubation, cells were measured on a FACSCalibur flow cytometer and analyzed after exclusion of cell doublets. All experiments were performed in three or more independent biological replicates.

### RNA Extraction and qPCR

Total RNA was isolated with Nucleospin miRNA kit (Macherey Nagel) and 5 µg of DNase-I treated total RNA was used to prepare cDNA using the Superscript-II Reverse Transcriptase kit (Invitrogen). Real-time qPCR was performed in triplicate using 1 µl cDNA (1∶20 dilution) with SYBR green (Invitrogen). Primer sequences used for qPCR are listed in Table S1. The qPCR data was first normalized to two housekeeping genes, *Sdha* and *Gapdh*, and represented as relative to one of the cell type. All experiments were performed in two or more independent biological replicates consisting of three technical replicates each.

### Whole Genome Microarray Analysis and Statistical Interpretation

Whole genome microarray analysis was performed essentially as described earlier [16]. Briefly, total RNA isolated from cells undergoing apoptosis that had been sorted by FACS was used to prepare cDNA. The Cy3- or Cy5-labeled cDNA was hybridized to Agilent Technologies 44K Mouse Whole Genome Microarrays (G4122A). Data intensities were extracted using the software ‘Feature Extraction 9.1′ (Agilent Technologies, Germany), and the raw microarray data were normalized with a non-linear loess regression method [21]. Differentially regulated genes were identified by ANOVA, and the resulting data were adjusted with the Benjamini-Hochberg method to control the False-Discovery-Rate [22]. All experiments were performed in three independent biological replicates.

**Figure 5:**
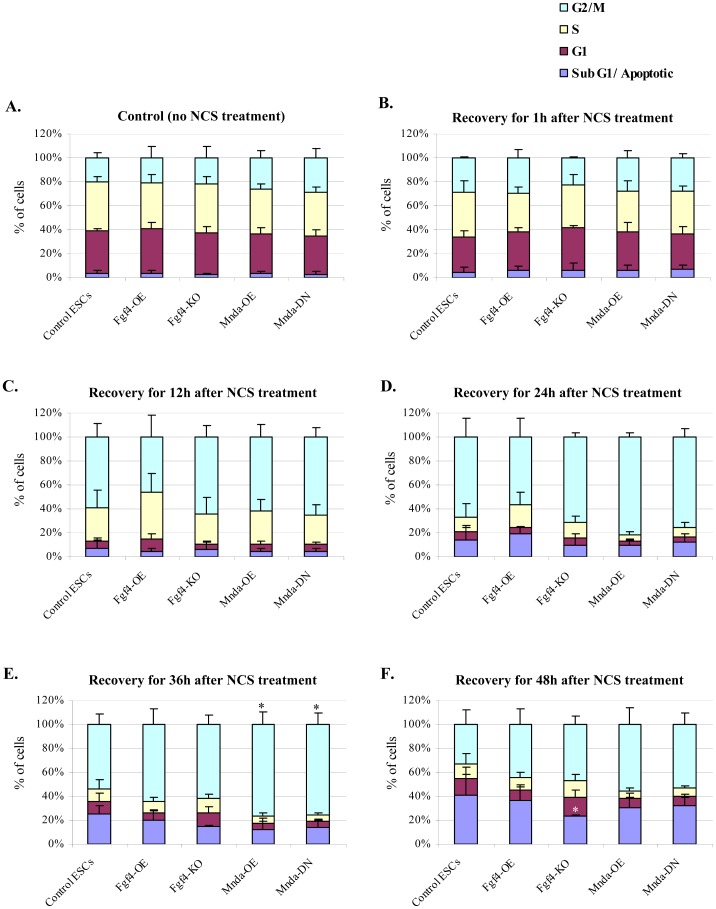
Role of *Fgf4* and *Mnda* during genotoxic stress response of ESCs. (**A**) Stacked bar graphs showing the percentage of cells at various stages of cell cycle (SubG1/Apoptotic, G1, S and G2/M) in untreated, Control ESCs – wildtype ESCs; Fgf4-OE – Fgf4 overexpression cells; Fgf4-KO – Fgf4 knockout cells; Mnda-OE – Mnda overexpression cells; Mnda-DN – Mnda downregulation cells. (**B–F**) Genotoxic stress was induced by treatment with NCS for 30 min followed by recovery for indicated time points and analyzed for cell cycle parameters in Control ESCs – wildtype ESCs; Fgf4-OE – Fgf4 overexpression cells; Fgf4-KO – Fgf4 knockout cells; Mnda-OE – Mnda overexpression cells; Mnda-DN – Mnda downregulation cells. The cell cycle data of three or more independent biological replicates were calculated and represented as a mean ±SD. The values which are statistically significant are indicated with asterisks (∗p<0.05).

### Teratoma Formation Assay

Teratoma formation assay was performed as previously described [23], [24]. Briefly, the indicated cell line (1×10^6^ cells) were injected subcutaneously into 8 to 10 weeks old female immunodeficient RAG2^−/−^cγc^−/−^ mice. Tumor growth was monitored weekly by palpation and size was recorded using linear calipers. Animals were sacrificed when a tumor diameter of 1 cm was reached. Autopsies were performed and tumor tissue was placed in phosphate-buffered 4% formalin for 16 h and then embedded in paraffin. For histological analysis, the specimens were stained with haematoxylin and eosin (HE).

### Statistical Analysis

All qPCR data for RNA expression analysis (two or more biological replicates) were calculated using delta-delta-Ct method. For statistical significance calculations, 2way ANOVA or Student’s t-test were used (GraphPad Prism 4.0).

## Results

### Apoptosis-related Gene Expression Profiling of ESCs and maGSCs

Genotoxic stress leads to the activation of p53-dependent mechanisms to either repair DNA damage or undergo apoptosis. The high sensitivity of ESCs to genotoxic stress is presumed to be due to their particular expression of pro-apoptotic and anti-apoptotic genes. To compare the apoptosis-related gene expression profiles of ESCs and maGSCs, we used OligoGEArray consisting of 112 genes implicated in the process of apoptosis (Fig. 1A). The comparison between undifferentiated ESCs and maGSCs of the 129Sv genetic background revealed similar gene expression patterns without any cell type-specific gene expression (Fig. 1B). The majority of the expressed genes are involved in the mitochondrial apoptotic pathway (intrinsic) and included no genes, such as *Fas* and *Fadd*, which are implicated in the extrinsic apoptotic pathway. Among all the analyzed caspases, only *Casp-2* was expressed at detectable level in both cell types. Quantification of the signal intensities using the OligoGEArray suite and scatterplot analysis revealed that, although many genes are equally expressed (below the fold difference threshold of 2) in ESCs and maGSCs, the pro-apoptotic gene *Bok* and three anti-apoptotic/tumor suppressor genes (*p53*, *Birc2*, and *Birc5*) showed significant upregulation in maGSCs (Fig. 1C). We verified the expression of apoptosis and anti-apoptosis genes in our previous global transcriptome comparison of undifferentiated ESCs and maGSCs [16], and found the upregulation of *Birc5*, but not other genes (Fig. S1A, E). Next, we compared the apoptosis-related gene expression profile of ESCs and maGSCs generated from Stra8-EGFP transgenic mice, hereafter referred as ESCs-Stra8 and maGSCs-Stra8, respectively. The gene expression profile of ESCs-Stra8 and maGSCs-Stra8 is identical to each other (Fig. 1D) and is also similar to those of ESCs and maGSCs of the 129Sv background (Fig. 1B). The quantification and subsequent scatterplot analysis showed the slight downregulation of pro-apoptotic genes, *Bnip3*, *Tnfrsf12a*, and *p53* in maGSCs-Stra8 (Fig. 1E). The opposing expression pattern of *p53* between two different genetic backgrounds of maGSCs (maGSCs-129Sv and maGSCs-Stra8) prompted us to verify these differences by more sensitive method, qRT-PCR. The qPCR analysis of *p53* as well as two other differentially expressed genes (*Bax* and *Nfkb1*), and two equally expressed control genes (*Atf5* and *Bok*) revealed no significant differences between maGSCs and ESCs of different backgrounds (Fig. S2).

We also analyzed the expression profile of pro-apoptotic and anti-apoptotic genes in ESCs and maGSCs that have been induced to differentiate for 20 days with retinoic acid (RA). The expression pattern of apoptosis-related genes is quite similar in both differentiated cell types (Fig. 2A) and is markedly distinct from the expression pattern of undifferentiated cells (Fig. 1B–E). The scatterplot analysis revealed the upregulation of *Nfkb1* and the downregulation of *Pycard* and *Bnip3* genes in differentiated maGSCs in comparison to differentiated ESCs (Fig. 2B). To verify these results, we analysed the global transcriptome data of differentiated ESCs and maGSCs [16], and found no obvious differences in the expression of apoptosis and anti-apoptosis genes (Fig. S1B, F). To highlight the differences between undifferentiated and differentiated cells and also to identify the genes that are specifically expressed in undifferentiated ESCs and maGSCs; we generated a heatmap of all OligoArray data sets (Fig. 2C). All undifferentiated cells possessed similar gene expression patterns and are clustered together, whereas differentiated cells possessed an expression pattern that is quite distinct and are clustered separately (Fig. 2C). Further analysis of the heatmap revealed strong and specific expression of *Mcl1*, *Bax*, *Hells*, and *Nfkb1* genes in undifferentiated ESCs and maGSCs, whereas the expression of these genes was highly downregulated or absent in their differentiated counterparts (Fig. 2C′). The expression of *p53*, *Api5*, *Birc2*, *Cradd*, and *Bid* genes was moderately high in undifferentiated pluripotent cells compared to RA-differentiated cells (Fig. 2C′). In agreement with these results, we found a significant downregulation of *Hells*, *Nfkb1*, and *p53* in our previous transcriptome data of differentiated ESCs and maGSCs [16] (Fig. S1C, D and G, H). In contrast to OligoGEArray results, we found few apoptotic as well as anti-apoptotic genes (*Dapk1*, *Ltbr*, *Xiap*, and *Bcl2l1*) to be upregulated in the transcriptome data of differentiated ESCs and maGSCs (Fig. S1C, D and G, H). However, these upregulated genes were not detectable in our OligoGEArray studies, suggesting their possible low expression levels which are difficult to detect using OligoGEArray.

### Citrinin-induced Apoptosis and Transcriptome Analysis of Early-apoptotic ESCs and maGSCs

The particular expression pattern of intrinsic apoptotic pathway-related genes led us to evaluate how both ESCs-129Sv and maGSCs-129Sv (hereafter referred as ESCs and maGSCs, respectively) react to the induction of apoptosis through the intrinsic pathway. Citrinin (CTN), a mycotoxin, is known to induce apoptosis through the intrinsic pathway in ESCs and to generate reactive oxygen species (ROS), which cause DNA damage and subsequent p53- induced apoptosis [25]. Because of these facts, CTN is a suitable compound to study mitochondrial as well as p53-mediated apoptosis. In this context, we induced apoptosis in ESCs and maGSCs with CTN (200 µM) for 24 h and analyzed the percentage of apoptotic cells by annexin-V and 7-AAD staining (Fig. 3A′, B′). After 24 h of CTN treatment, both ESCs and maGSCs had lost the characteristic colony morphology and appeared as typical apoptotic cells (Fig. 3A, B). Flow cytometric analysis after annexin-V and 7-AAD staining revealed a substantial presence of early-apoptotic cells (∼35% of the total cell population, annexin-V^+ve^/7-AAD^−ve^) in CTN-treated ESCs and maGSCs, but only ∼8–15% of un-treated and DMSO control cells displayed an early-apoptotic phenotype (Fig. 3A′, B′). The percentage of late apoptotic (annexin-V^+ve^/7-AAD^+ve^) and viable (annexin-V^−ve^/7-AAD^−ve^) cells were also significantly similar between CTN-treated ESCs and maGSCs. Overall, these results indicate that maGSCs, like ESCs, are reactive to CTN and undergo apoptosis at a similar rate.

The intrinsic-pathway related gene expression pattern seen in our OligoArray suggests that this pathway is constitutively active in pluripotent cells and might play a role in resistance to genotoxicity. Because, CTN is also known to induce DNA damage through the generation of ROS and the subsequent activation of the intrinsic apoptotic pathway, we performed transcriptome analysis using early-apoptotic cells as outlined (Fig. 3C) to identify the factors that are responsible for maintaining genomic integrity. Transcriptome analysis of early-apoptotic ESCs and maGSCs revealed that the majority of genes were equally expressed in both cell types (∼94% similarity), but 1257 (∼6%) genes were differentially expressed (Fig. 3D). Several of the pluripotency marker genes expression was slightly downregulated in early-apoptotic cells transcriptome compared to our previously described transcriptome of undifferentiated ESCs and maGSCs [16] (Table S2). Similar analysis for differentiation marker genes of three germ layers revealed no overt changes in their expression, except for downregulation of *Vimentin*, a mesodermal marker gene (Table S3). Comparison of early-apoptotic ESCs transcriptome with transcriptome of undifferentiated ESCs [16] showed ∼80% similarity, indicating that only ∼20% genes are differentially regulated in early-apoptotic cells (Fig. S3A). Similarly, early-apoptotic maGSCs showed ∼67% similarity to their undifferentiated counterparts and ∼33% differentially regulated genes (Fig. S3B). Further comparison of differentially regulated genes in early-apoptotic ESCs and maGSCs showed ∼44% of commonly upregulated and ∼55% commonly downregulated genes (Fig. S3C, D, and Tables S4 & S5). The functional gene ontology (GO) analysis revealed that genes associated with several biological processes such as signal transduction, cell cycle processes, response to stress, DNA damage, and DNA repair are upregulated in early-apoptotic cells compared to wild-type cells (Table 1). Interestingly, genes associated with biological processes such as regulation of transcription, oxidation reduction, intracellular signalling cascade, cell proliferation, cell death, cell adhesion are downregulated in early-apoptotic cells (Table 2). The principle component analysis (PCA) of the transcriptome of early-apoptotic cells and previously described transcriptomes of undifferentiated and differentiated cells [16] clearly showed the clustering of early-apoptotic ESCs and maGSCs, which are quite distant from their undifferentiated and differentiated counterparts (Fig. 3E). Similar results were obtained when we analyzed the transcriptome data using a hierarchical clustering model (Fig. S4).

### Identification of New Pluripotent Cell-specific and Apoptosis-related Candidate Genes

The transcriptome analysis of early-apoptotic ESCs and maGSCs facilitated the identification of new candidate genes that might be specifically expressed in pluripotent cells and might play a role in genotoxicity-triggered cell death. We focused on genes with either direct or indirect role in apoptosis and are highly modulated in early-apoptotic cells (Tables S4 and S5), to characterize their role in response to genotoxicity. Further, we performed quantitative real-time PCR (qPCR) for the selected candidate genes and found downregulated candidate genes, *Matrix metalloproteinase-10* (*Mmp10*) and *Fibroblast growth factor 4* (*Fgf4*) to be specifically expressed in pluripotent cells but not in any of the analyzed adult mouse tissues (Fig. 4A). Another downregulated candidate gene that we selected is *Myeloid cell nuclear differentiating agent* (*Mnda*) which bears a death domain with an unknown function. Although *Mnda* is expressed in heart and liver, apart from pluripotent cells, it is expressed in no other mouse tissue (Fig. 4A). Expression analysis of other downregulated candidate genes (*Adm*, *Fbln2*, and *Hist1h1b*) revealed ubiquitous expression (data not shown), hence omitted from further analysis. Similarly, we have validated the upregulation of three upregulated candidate genes (*Hist2h2be*, *Hspa1*, and *Mia2*) using qRT-PCR (Fig. S5), however, these genes showed ubiquitous tissue expression (data not shown). Hence, these genes were not considered for further analysis. Based on the above results, we choose *Fgf4* and *Mnda* for further analysis, because of their known direct or indirect association with apoptosis [26], [27], and performed qPCR to validate the downregulation seen in the transcriptome. The qPCR data further confirmed the downregulation of Fgf4 and Mnda in early-apoptotic cells (Fig. 4B, C).

### Role of *Fgf4* in Response to Apoptosis and Genotoxicity Induction

To study the role of *Fgf4* in response to induced genotoxicity and apoptosis, we generated an ESC line with stable *Fgf4* overexpression (*Fgf4*-OE) that expressed *Fgf4* at levels approximately 15-fold higher than those found in control cells (Fig. S6A) and also used a previously described *Fgf4^−/−^* ESC line (*Fgf4*-KO) [20]. Both *Fgf4*-OE and *Fgf4*-KO cell lines showed normal colony morphology and proliferation under standard ESC culture conditions (data not shown). Moreover, when induced to differentiate for 12 days with RA treatment, both cell types demonstrated differentiation potential comparable to control cells, as assessed by qPCR of pluripotency and differentiation marker genes (Fig. S7A and B). Previously, *Fgf4*-KO cells were shown to differentiate into all three germ layers in teratoma formation assay [20]. We tested the teratoma formation potential of *Fgf4*-OE cells and found cell types from all three germ layers (Fig. S7C), suggesting that the alteration of *Fgf4* expression has no overt effect on their differentiation potential. We challenged both *Fgf4*-KO and *Fgf4*-OE cells with CTN for 12 h and 24 h, and assayed for survival with annexin-V and 7-AAD staining. Analysis of these results indicated that both *Fgf4*-OE and *Fgf4*-KO cells responded to CTN in a similar manner with no noticeable differences from the wild-type cells response (Fig. 4D–F).

To analyze the DNA damage response in *Fgf4*-OE and *Fgf4*-KO, we induced DNA damage with neocarzinostatin (NCS), a known, potent inducer of DSBs, similar to γ-irradiation, in various cell types. Currently, there are no reports of NCS treatment on ESCs or other pluripotent cell lines. To verify the DSBs induction in ESCs, we stained the control and NCS treated ESCs with γ-H2A.X, a marker for DSBs (Fig. S8). Analysis of these results indicated the presence of a weak but significant amount of γ-H2A.X staining in control cells and higher signal intensities in NCS-treated cells (Fig. S8). The γ-H2A.X foci are gradually decreased by 24 h of recovery (Fig. S8) indicating DNA damage induction by NCS and subsequent activation of DNA damage repair machinery. Treatment of maGSCs with NCS revealed DNA damage induction and repair with similar pattern like ESCs (Fig. S8), suggesting the existence of similar DNA damage response mechanisms in both cell types. Thereafter, we treated *Fgf4*-OE, *Fgf4*-KO, and wild-type control ESCs with NCS and analyzed cell cycle parameters at various time points of recovery (Fig. 5A–F and Fig. S9). We found that control ESCs displayed a G2/M arrest, as previously shown with γ-irradiation [11] (Fig. 5A–F). Similarly, *Fgf4*-KO and *Fgf4*-OE cells also displayed a G2/M arrest (Fig. 5A–F). Approximately 35–40% of control cells were subG1/apoptotic by 48 h (Fig. 5F). Strikingly, fewer *Fgf4*-KO cells were apoptotic at 48 h (∼20%), whereas approximately 35–40% of both control and *Fgf4*-OE cells were apoptotic at this time point (Fig. 5F). The representative images of flow cytometry measurements at various time points are provided in Fig. S9.

### Role of Mnda in Response to Apoptosis and Genotoxicity Induction

To study the role of *Mnda* in response to genotoxicity- and apoptosis-inducing stimuli, we generated a stable *Mnda*-overexpressing cell line (*Mnda*-OE), in which *Mnda* is expressed at levels ∼3-fold higher than in wild-type cells (Fig. S6B). We also generated a cell line (*Mnda*-DN), in which *Mnda* is downregulated through stable expression of shRNA against *Mnda*, resulting in approximately 90% downregulation (Fig. S6B). These two cell lines displayed normal colony morphology and proliferation under standard ESC culture conditions (data not shown) and also differentiated to all three germ layers when treated with either RA or assayed for teratoma formation (Fig. S7A–C). We challenged *Mnda*-DN and *Mnda*-OE cells with CTN for 12 h and 24 h, and analyzed for the survival rate. No overt differences in cell survival rates were observed between control, *Mnda*-OE, and *Mnda*-DN cells (Fig. 4D, G, H).

Additionally, we treated *Mnda*-OE and *Mnda*-DN cells with NCS and analyzed the cell cycle parameters at various time points (Fig. 5A–F and Fig. S9). Cell cycle analysis indicated that a majority (∼80%) of both *Mnda*-OE and *Mnda*-DN cells undergo G2/M arrest by 24 h and that this arrest persisted even after 36 h of recovery (Fig. 5D, E). Finally, we did not observe any differences in the percentage of apoptotic cells between *Mnda*-OE, *Mnda*-DN, and control cells (Fig. 5F). The representative images of flow cytometry measurements at various time points are provided in Fig. S9.

## Discussion

Several studies have reported that multipotent germline stem cells (mGSCs) that are morphologically and functionally similar to ESCs can be generated from SSCs of neonatal and adult mouse testis, suggesting that SSCs have the ability to give rise to pluripotent cells *in vitro* [14], [28], [29], [30], [31], [32]. mGSCs from adult mouse testis, termed multipotent adult germline stem cells (maGSCs), were first generated and described in 2006 by our group [14]. To further evaluate the pluripotent cell characteristics of maGSCs, we extensively studied maGSCs alongside ESCs and demonstrated the similarities between these two cell types [15], [16], [17], [18], [19]. In the present study, we investigated the apoptosis-related gene expression profiles and also the cellular responses to apoptosis induction. Further, we selected two candidate genes, namely *Fgf4* and *Mnda*, and analyzed their roles in apoptosis or genotoxicity responses in pluripotent cells.

ESCs are characterized by self-renewal, a high rate of proliferation, a characteristic short cell cycle, and the potential to differentiate into all cell lineages of the organism. It is essential to understand how ESCs maintain genome stability and integrity during this rapid proliferation. It has been suggested that ESCs might have evolved with conserved and precise mechanisms to prevent the accumulation of mutations. The existence of such protective mechanisms is further supported by the following evidence: (1) the mutation and somatic recombination frequencies of mouse ESCs are 100-fold lower than in adult somatic cells (fibroblasts) [9], (2) human and mouse ESCs bear a very efficient antioxidant defense against DNA damage mediated by ROS [33], [34], and (3) ESCs are hypersensitive to genotoxicity and can undergo apoptosis without the activation of cell cycle checkpoints, leading to the effective elimination of defective cells [6].

To evaluate the pluripotent cell-specific, apoptosis-related properties of maGSCs, we analyzed the expression of apoptosis-related genes and found an expression profile similar to ESCs. Both cell types displayed the expression of genes involved in the intrinsic apoptotic pathway, suggesting a strong similarity between undifferentiated ESCs and maGSCs. Interestingly, we could not detect the expression of genes involved in extrinsic pathway of apoptosis (death receptors and their ligands) suggesting the inactivity or weak activity of this pathway in both pluripotent cell types. Consistent with this data, an earlier study showed the expression of *Fas* receptor only after inducing apoptosis through O6-methylguanine (O6 MeG) [35]. Further, we compared the gene expression profile in RA-differentiated ESCs and maGSCs to evaluate the patterns and similarities of apoptotic genes during differentiation. In agreement with our previous findings from gene expression analysis of RA-differentiated cells [16], both differentiated ESCs and maGSCs possessed an apoptosis-related gene expression profile similar to each other but in sharp contrast to that of undifferentiated cells. The expression of many anti-apoptotic and pro-apoptotic genes are downregulated in RA-differentiated cells, indicating that expression of these genes is specific to pluripotent cells. As ESCs are known to be highly sensitive to genotoxic stress and effectively eliminate damaged cells through apoptosis, the specific expression of these anti-apoptotic and pro-apoptotic genes may suggest a role in the elimination of defective cells.

The transcriptome analysis of early-apoptotic cells led to the identification of *Fgf4* and *Mnda* as highly downregulated genes that might play a role in mitochondrial or p53-mediated apoptotic response. Fgf4 belongs to the FGF superfamily of proteins and is involved in various stages of embryonic development [36]. Although *Fgf4* is highly expressed in ESCs relative to differentiated cells, alteration of its expression in undifferentiated ESCs has only slight effects on survival and morphology [20]. *Fgf4*-deficient ESCs show reduced survival of differentiated cells and this could be reversed by the addition of Fgf4 to the culture medium, suggesting that Fgf4 plays an essential role in differentiation [20]. Because the role of Fgf4 during induced apoptosis or genotoxicity in undifferentiated ESCs had not yet been explored, we induced apoptosis or genotoxicity in *Fgf4*-overexpressing and knock-out (*Fgf4*-OE and *Fgf4*-KO, respectively) ESCs. Interestingly, *Fgf4*-KO cells appeared to be partially protected against DNA damage-induced apoptosis, whereas *Fgf4*-OE cells did not display any abnormalities. These results are in contrast to previous findings, which showed that induced expression of Fgf4 functions as an anti-apoptotic factor of male germ cells and protects them from hyperthermia-induced apoptosis [26]. These contrasting results could partly be due to different cell types or due to the resistance of *Fgf4*-KO cells to differentiation. It has been reported that ESCs with DNA damage can induce differentiation by p53-dependent suppression of *Nanog* that leads to the effective elimination of those differentiated (Nanog negative) cells by p53-dependent cell cycle arrest and apoptosis [13]. It is interesting to note that the deficiency of Fgf4 blocks differentiation and increases cell survival [37], [38]. Hence, we suggest that *Fgf4*-KO cells might be resistant to DNA damage-induced differentiation (note: not a RA induced differentiation) and subsequent apoptosis by p53-dependent mechanisms. These results are in parallel with the observed downregulation of *Fgf4* during early-apoptosis, possibly to maintain their survival. It has been reported that the inhibition of Fgf/Erk signalling pathways can contribute to the derivation and maintenance of ‘naïve’ pluripotent stem cells [39], [40]. In wake of our observations, it remains to be investigated that whether *Fgf4* deficient/blocked pluripotent cells carry more DNA damage burden and chromosomal aberrations or have efficient DNA damage repair machinery to maintain the pluripotency.

Mnda is a member of the interferon (IFN)-regulated 200 family of proteins that contain a partially conserved 220-amino acid domain, and is thought to interact specifically with other transcriptional regulators [41]. The members of this family also contain a pyrin domain at the amino-terminus, which is proposed to function in programmed cell death and inflammation [42]. Moreover, several proteins of this family, including human MNDA (hMNDA), were shown to promote programmed cell death in several experimental conditions [43], [44], [45], [46], [47]. Until now, the expression of *hMNDA* was reported only in hematopoietic cells [44]. Moreover, the expression of *hMNDA* is most significantly downregulated in myelodysplastic syndrome (MDS), in which elevated levels of apoptosis are detected in granulocyte-macrophage progenitors [27]. Since the expression pattern and the role of mouse Mnda is not yet known and it is highly downregulated during CTN-induced apoptosis, we sought to analyze the function of this protein in responses to genotoxicity. Unfortunately, we did not observe any significant differences in protection or sensitivity to apoptosis between control and either *Mnda*-OE or *Mnda*-DN cells when apoptosis was induced through CTN. On the other hand, induction of DNA damage in *Mnda*-OE and *Mnda*-DN cells exhibited prolonged G2/M arrest. These results indicate that the expression level of *Mnda* in ESCs is critical to respond against DNA damage. The detection of similar apoptosis levels at 48 h between *Mnda*-altered cells and control cells is elusive and remains to be investigated.

Furthermore, the functional GO analysis of differentially expressed genes in early-apoptotic cells revealed the activation of stress response and DNA damage repair pathways, and downregulation of cell death pathways, suggesting the activation of cell survival mechanisms during early-apoptotic events. Taken together, the detected significant differences after DNA damage induction, but not during apoptosis induction, in ESCs with altered expression of *Fgf4* or *Mnda*, suggest that these genes play an essential role in maintaining genomic integrity and regulating cell survival in the very early-stages of induced apoptosis before the cascade of the intrinsic pathway becomes activated.

## Supporting Information

Figure S1 **Apoptosis and anti-apoptosis gene expression profiles in undifferentiated and differentiated ESCs and maGSCs transcriptomes.** Scatterplots showing the expression pattern of apoptosis (A–D), and anti-apoptosis (E–H) genes in previously described transcriptome data of undifferentiated ESCs and maGSCs of 129Sv-genetic background and their respective RA-treated differentiated counterparts. The expression of *Birc5* was upregulated whereas the expression of *Casp12*, *Casp6*, *Birc3*, *Cflar*, and *Bnip3* was downregulated in undifferentiated maGSCs compared to ESCs (A, E), No genes were found to be differentially expressed between differentiated ESCs and maGSCs (B, F). Expression of several apoptosis as well as anti-apoptosis genes was downregulated in differentiated ESCs (C, G), whereas *Ltbr*, *Dapk1*, and *Bcl2l1* showed upregulation in differentiated ESCs (C, G). Expression of several apoptosis as well as anti-apoptosis genes was downregulated in differentiated maGSCs (D, H), whereas *Casp12*, *Ltbr*, *Xiap2*, *Apaf1*, and *Dapk1* showed upregulation in differentiated ESCs (D, H).
(TIF)

Figure S2 **Validation of differentially expressed genes of OligoGEArray.** The bar graph showing the expression of two equally expressed genes (*Atf5* and *Bok*) and three differentially expressed genes (*p53*, *Bax*, and *Nfkb1*) in undifferentiated ESCs and maGSCs of 129Sv- and Stra8-genetic backgrounds.
(TIF)

Figure S3 **Comparison of undifferentiated, and early-apoptotic ESCs and maGSCs transcriptomes.** Venn diagram showing the comparison of early-apoptotic (early-apo) and undifferentiated (Wt) cells transcriptomes of ESCs **(A)** and maGSCs **(B)**. Venn diagram showing the upregulated genes in early-apoptotic ESCs and maGSCs and their similarities and differences **(C)**. Venn diagram showing the downregulated genes in early-apoptotic ESCs and maGSCs and their similarities and differences **(D)**.
(TIF)

Figure S4 **Hierarchical clustering of undifferentiated, differentiated, and early-apoptotic ESCs and maGSCs transcriptomes.** The clustering showing the similarities within three replicates of early-apoptotic ESCs and maGSCs as well as undifferentiated and differentiated cell types. The transcriptomes of early-apoptotic cells is again clustered together and is distinct from both undifferentiated and differentiated cells transcriptomes.
(TIF)

Figure S5 **Validation of early-apoptotic cells transcriptome data.** The qPCR data confirming the upregulation of *Hspa1a* **(A)**, *Hist2h2be* **(B)**, and *Mia2* **(C)** in early-apoptotic ESCs and maGSCs (ESCs_apo and maGSCs_apo, respectively).
(TIF)

Figure S6 **Expression analysis of overexpressing, knockdown, and knockout cell lines. (A)** Real-time qPCR analysis showing the expression of *Fgf4* in control, *Fgf4*-OE, and *Fgf4*-KO cells. **(B)** Real-time qPCR analysis showing the expression of *Mnda* in control, *Mnda*-OE, and *Mnda*-DN cells.
(TIF)

Figure S7 **Differentiation potential of overexpression and knockdown cell lines. (A)** Heatmap representing the qPCR data of pluripotency marker genes, *Nanog* and *Zfp206* expression in undifferentiated and differentiated control ESCs, *Fgf4*-OE, *Fgf4*-KO, *Mnda*-OE, and *Mnda*-DN cell lines. The expression of *Nanog* and *Zfp206* was reduced or absent in differentiated cells as expected. **(B)** Heatmap representing the qPCR data of differentiation marker genes, *Hnf4*, *Vimentin*, and *Nestin* expression in undifferentiated and differentiated control ESCs, *Fgf4*-OE, *Fgf4*-KO, *Mnda*-OE, and *Mnda*-DN cell lines. Expression of *Hnf4*, *Vimentin*, and *Nestin* was upregulated, indicating differentiation. **(C)** Tumors obtained from immunodeficient mice after injection of *Fgf4*-OE, *Mnda*-OE, and *Mnda*-DN ESCs were HE stained and identified as teratomas. The representative cell or tissue types for all three germ layers were indicated with arrowheads.
(TIF)

Figure S8 **Analysis of NCS induced DNA damage in ESCs and maGSC.** Wild-type ESCs **(A)** or maGSCs **(B)** were treated with NCS for 30 min and allowed to recover for 24 h. Immunostaining at indicated time points using γH2A.X (green) indicated the strong induction of DSBs by 1 h and gradual disappearance by 24 h, indicating DNA repair. Inset showing the γH2A.X foci of the enlarged region (dotted line). DAPI (blue) was used to stain the nucleus.
(TIF)

Figure S9 **Cell cycle analysis parameters and patterns of DNA damage induced cells. (A)** Representative images showing the gating parameters used to exclude doublets from the analysis. **(B)** Representative images showing the cell cycle patterns in control or NCS treated and recovered cells at indicated time points.
(TIF)

Table S1 **Primers used in this study.**
(DOC)

Table S2 **Expression of pluripotency marker genes in early-apoptotic ESCs and maGSCs compared to their undifferentiated counter parts.**
(XLS)

Table S3 **Expression of differentiation marker genes in early-apoptotic ESCs and maGSCs compared to their undifferentiated counter parts.**
(XLS)

Table S4 **List of upregulated genes in early-apoptotic ESCs and maGSCs.**
(XLS)

Table S5 **List of downregulated genes in early-apoptotic ESCs and maGSCs.**
(XLS)
