# Supplementary figures and images for: Apoptosis-Related Gene Expression Profiles of Mouse ESCs and maGSCs: Role of Fgf4 and Mnda in Pluripotent Cell Responses to Genotoxicity
Source: PLoS One. 2012 Nov 7;7(11):e48869. doi: 10.1371/journal.pone.0048869 (PMC3492253; doi:10.1371/journal.pone.0048869)

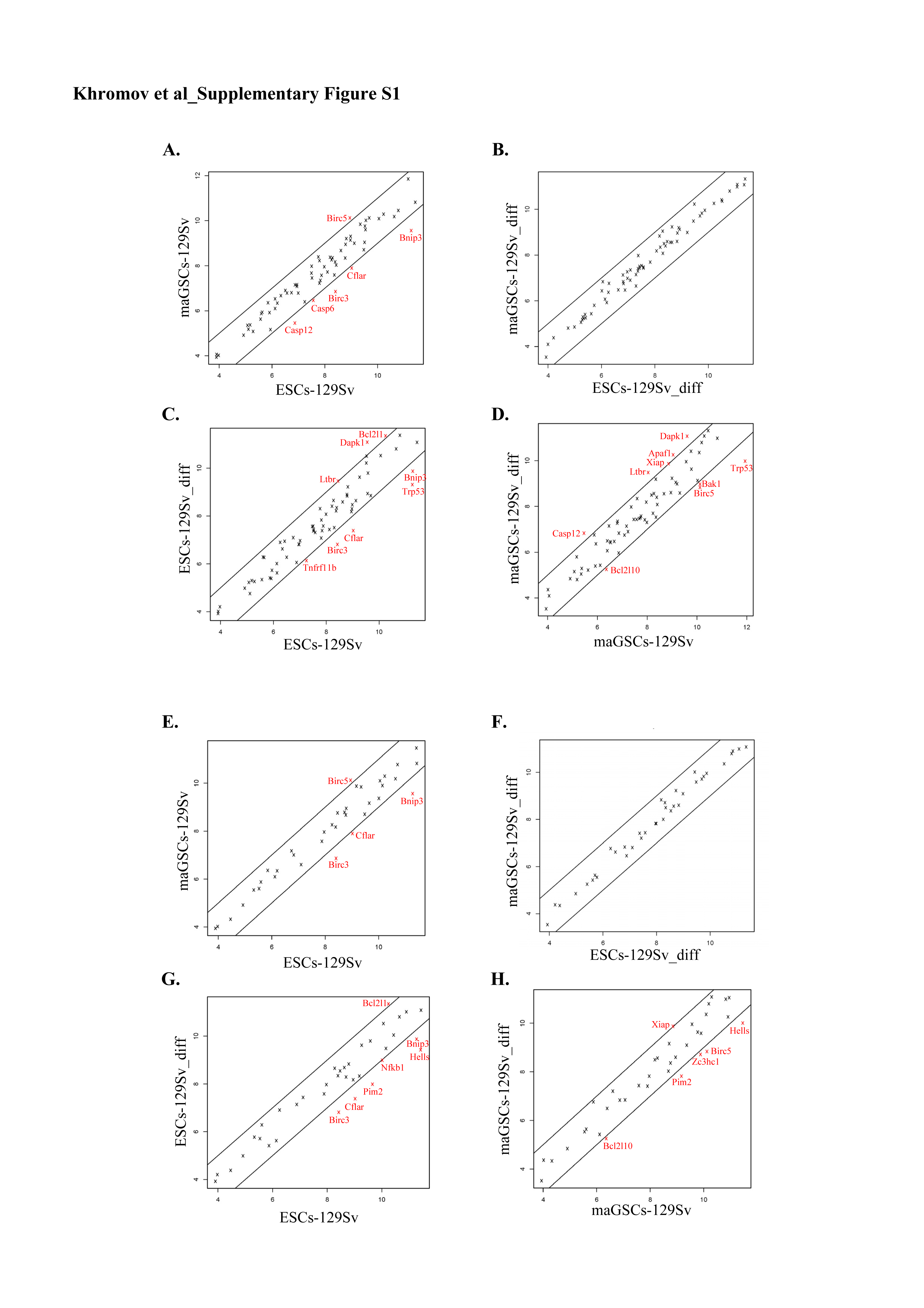

Supplement: Figure S1 — Apoptosis and anti-apoptosis gene expression profiles in undifferentiated and differentiated ESCs and maGSCs transcriptomes. Scatterplots showing the expression pattern of apoptosis (A–D), and anti-apoptosis (E–H) genes in previously described transcriptome data of undifferentiated ESCs and maGSCs of 129Sv-genetic background and their respective RA-treated differentiated counterparts. The expression of Birc5 was upregulated whereas the expression of Casp12, Casp6, Birc3, Cflar, and Bnip3 was downregulated in undifferentiated maGSCs compared to ESCs (A, E), No genes were found to be differentially expressed between differentiated ESCs and maGSCs (B, F). Expression of several apoptosis as well as anti-apoptosis genes was downregulated in differentiated ESCs (C, G), whereas Ltbr, Dapk1, and Bcl2l1 showed upregulation in differentiated ESCs (C, G). Expression of several apoptosis as well as anti-apoptosis genes was downregulated in differentiated maGSCs (D, H), whereas Casp12, Ltbr, Xiap2, Apaf1, and Dapk1 showed upregulation in differentiated ESCs (D, H). (TIF) [file pone.0048869.s001.tif]

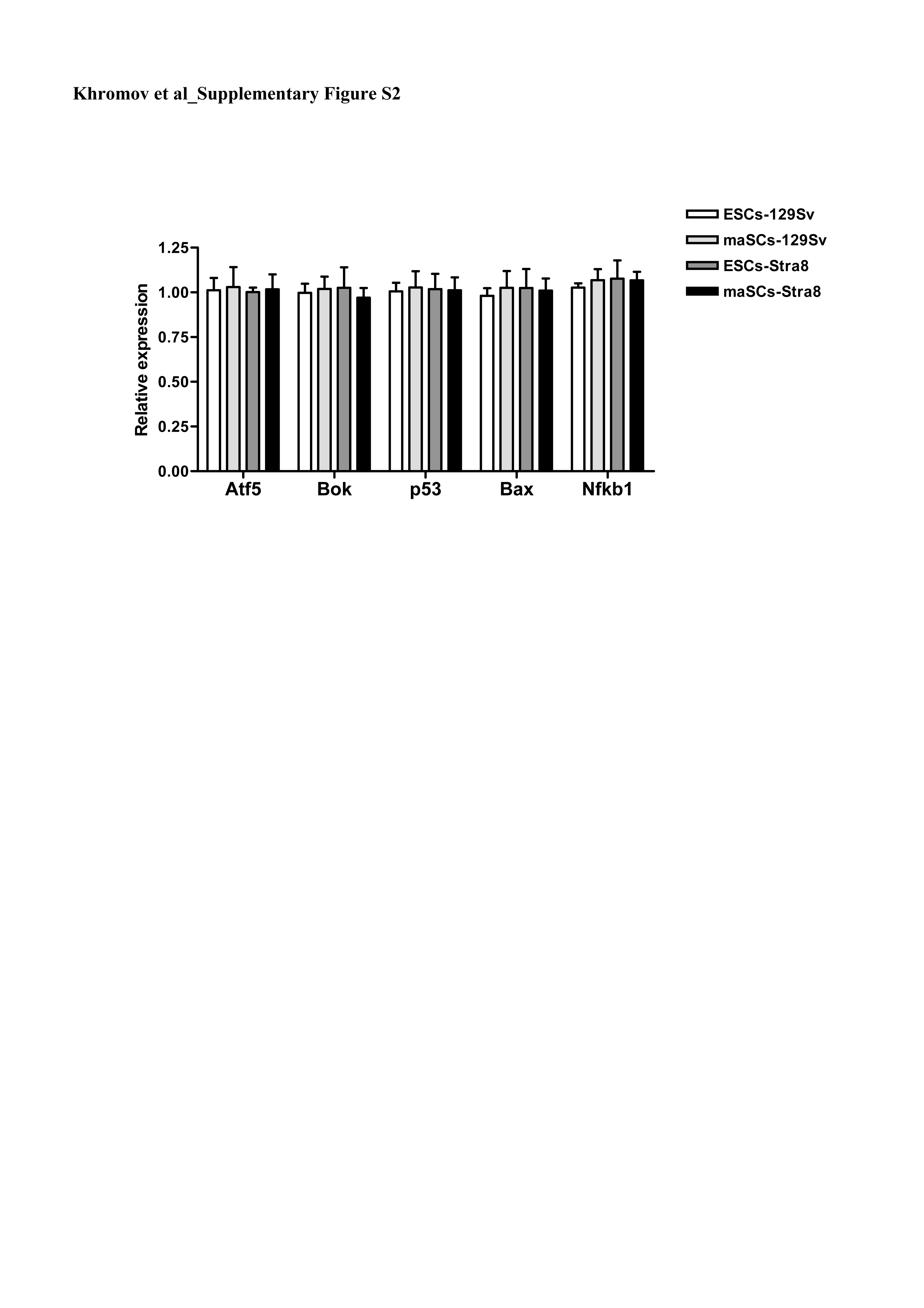

Supplement: Figure S2 — Validation of differentially expressed genes of OligoGEArray. The bar graph showing the expression of two equally expressed genes (Atf5 and Bok) and three differentially expressed genes (p53, Bax, and Nfkb1) in undifferentiated ESCs and maGSCs of 129Sv- and Stra8-genetic backgrounds. (TIF) [file pone.0048869.s002.tif]

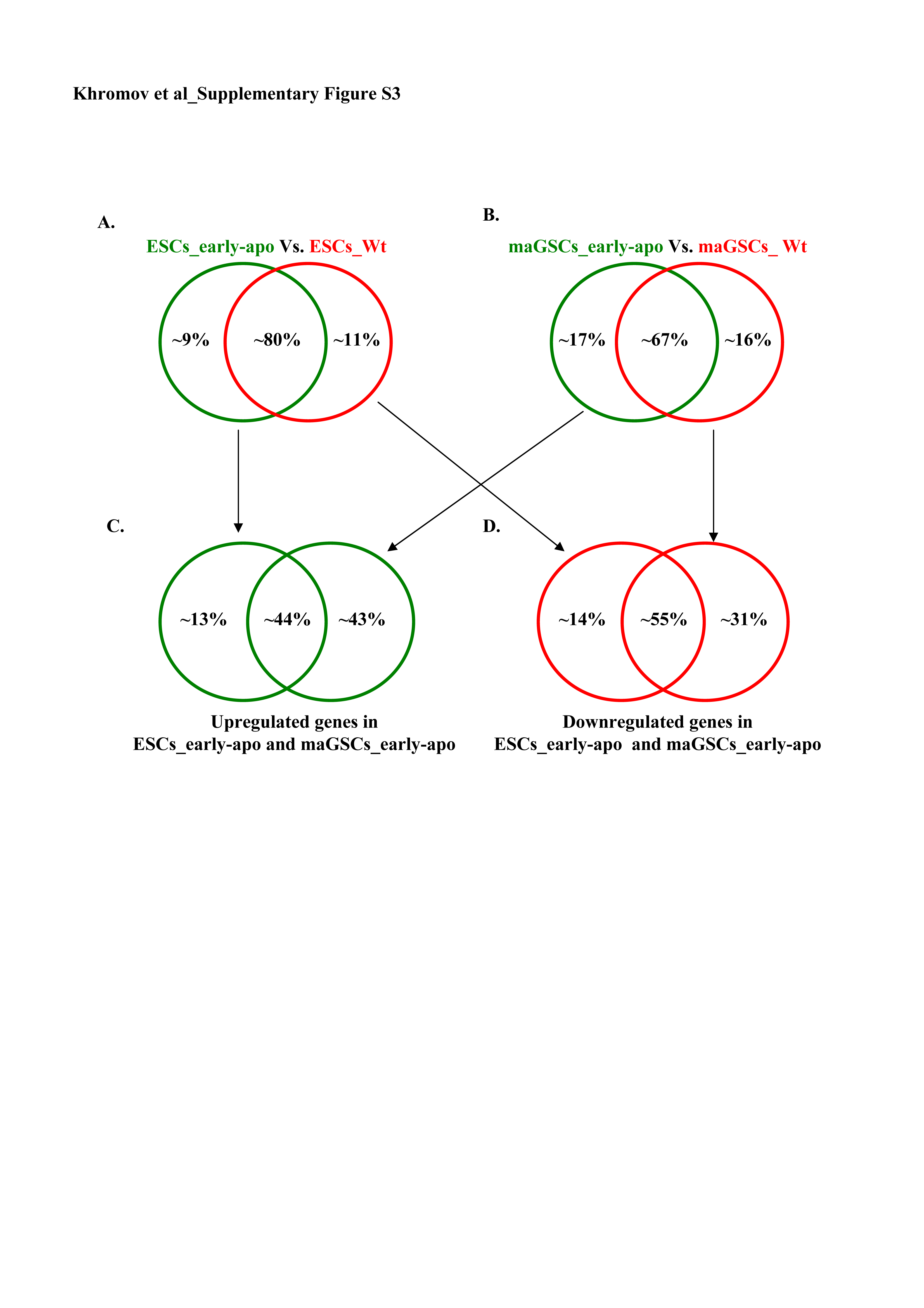

Supplement: Figure S3 — Comparison of undifferentiated, and early-apoptotic ESCs and maGSCs transcriptomes. Venn diagram showing the comparison of early-apoptotic (early-apo) and undifferentiated (Wt) cells transcriptomes of ESCs (A) and maGSCs (B). Venn diagram showing the upregulated genes in early-apoptotic ESCs and maGSCs and their similarities and differences (C). Venn diagram showing the downregulated genes in early-apoptotic ESCs and maGSCs and their similarities and differences (D). (TIF) [file pone.0048869.s003.tif]

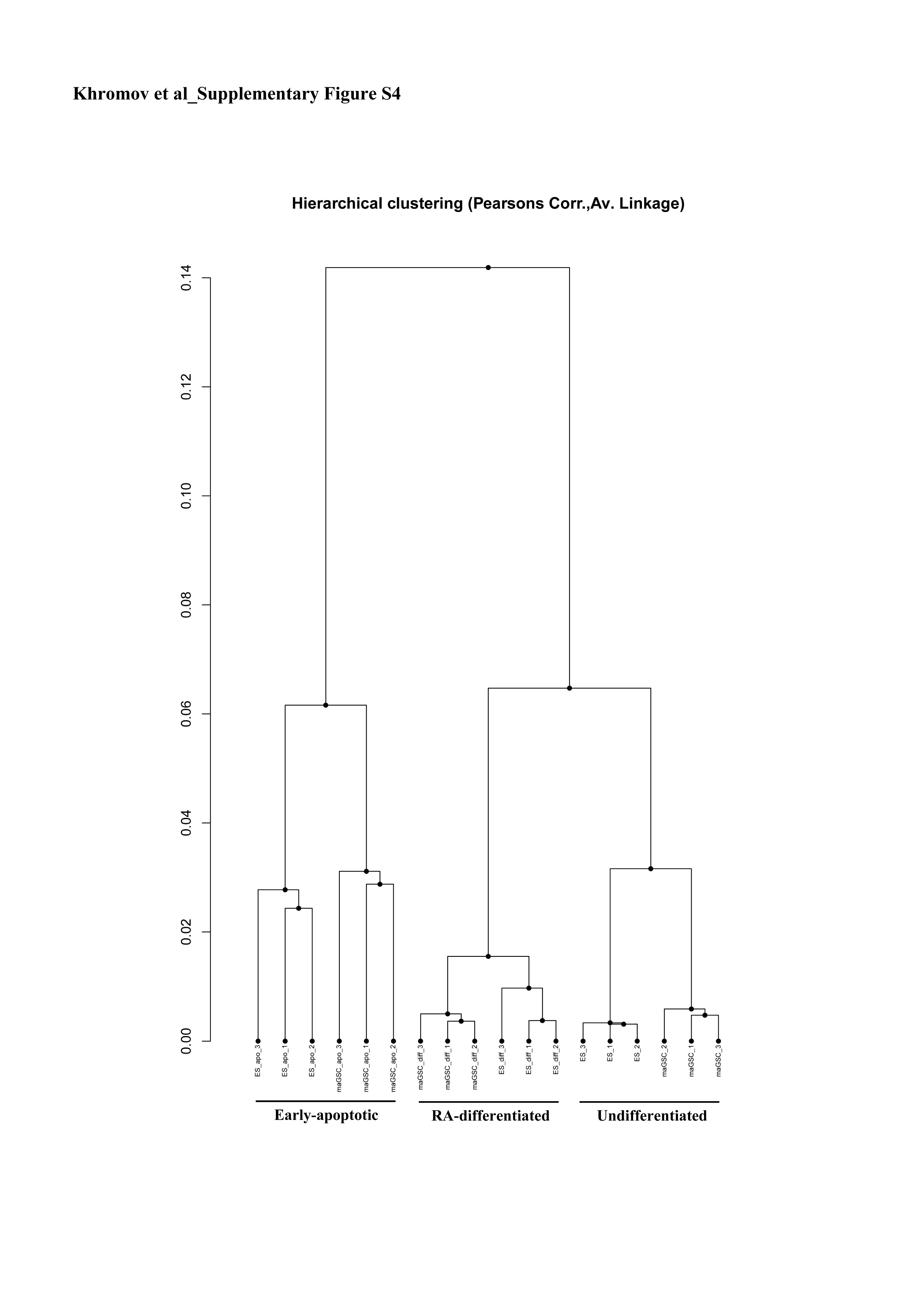

Supplement: Figure S4 — Hierarchical clustering of undifferentiated, differentiated, and early-apoptotic ESCs and maGSCs transcriptomes. The clustering showing the similarities within three replicates of early-apoptotic ESCs and maGSCs as well as undifferentiated and differentiated cell types. The transcriptomes of early-apoptotic cells is again clustered together and is distinct from both undifferentiated and differentiated cells transcriptomes. (TIF) [file pone.0048869.s004.tif]

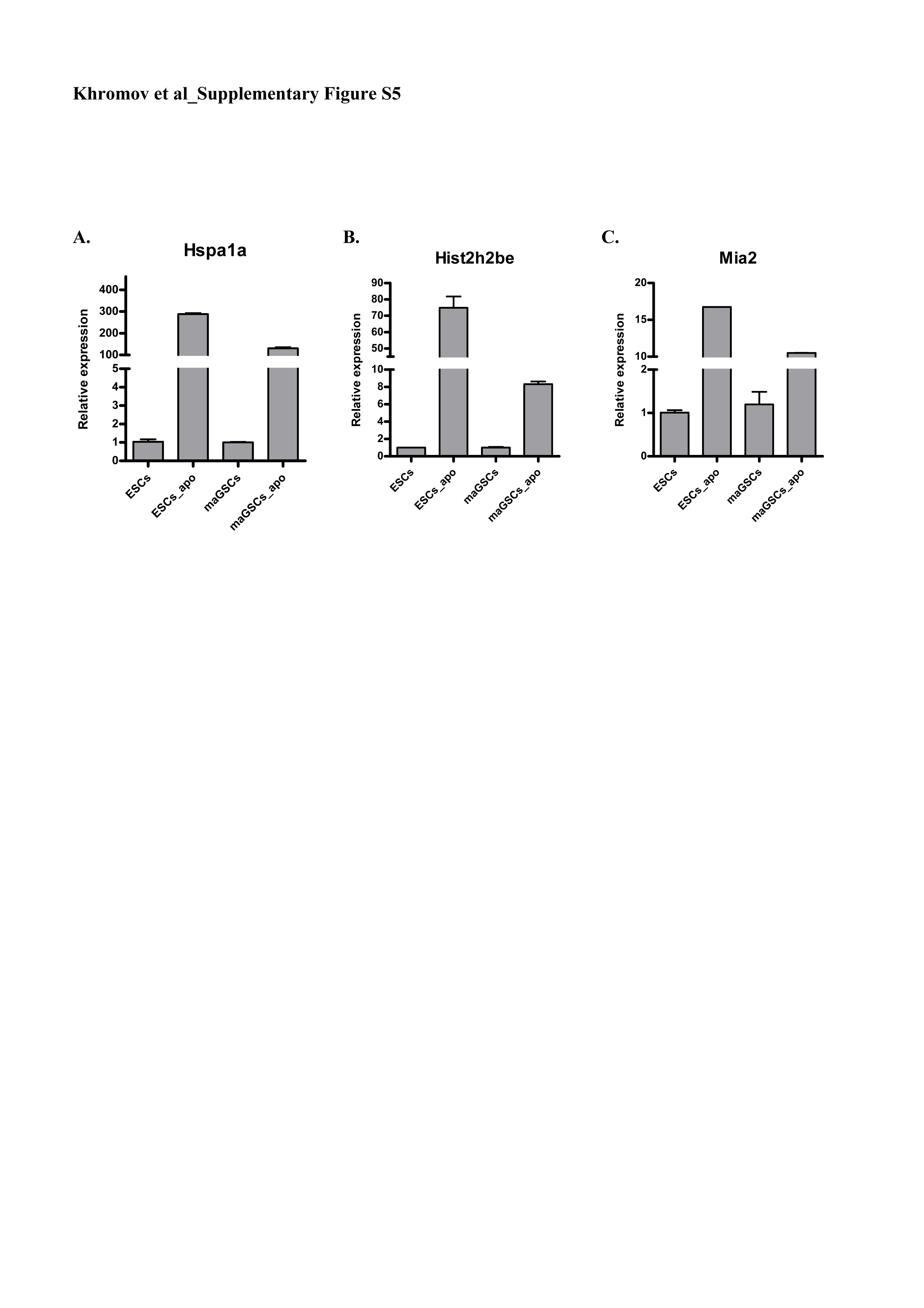

Supplement: Figure S5 — Validation of early-apoptotic cells transcriptome data. The qPCR data confirming the upregulation of Hspa1a (A), Hist2h2be (B), and Mia2 (C) in early-apoptotic ESCs and maGSCs (ESCs_apo and maGSCs_apo, respectively). (TIF) [file pone.0048869.s005.tif]

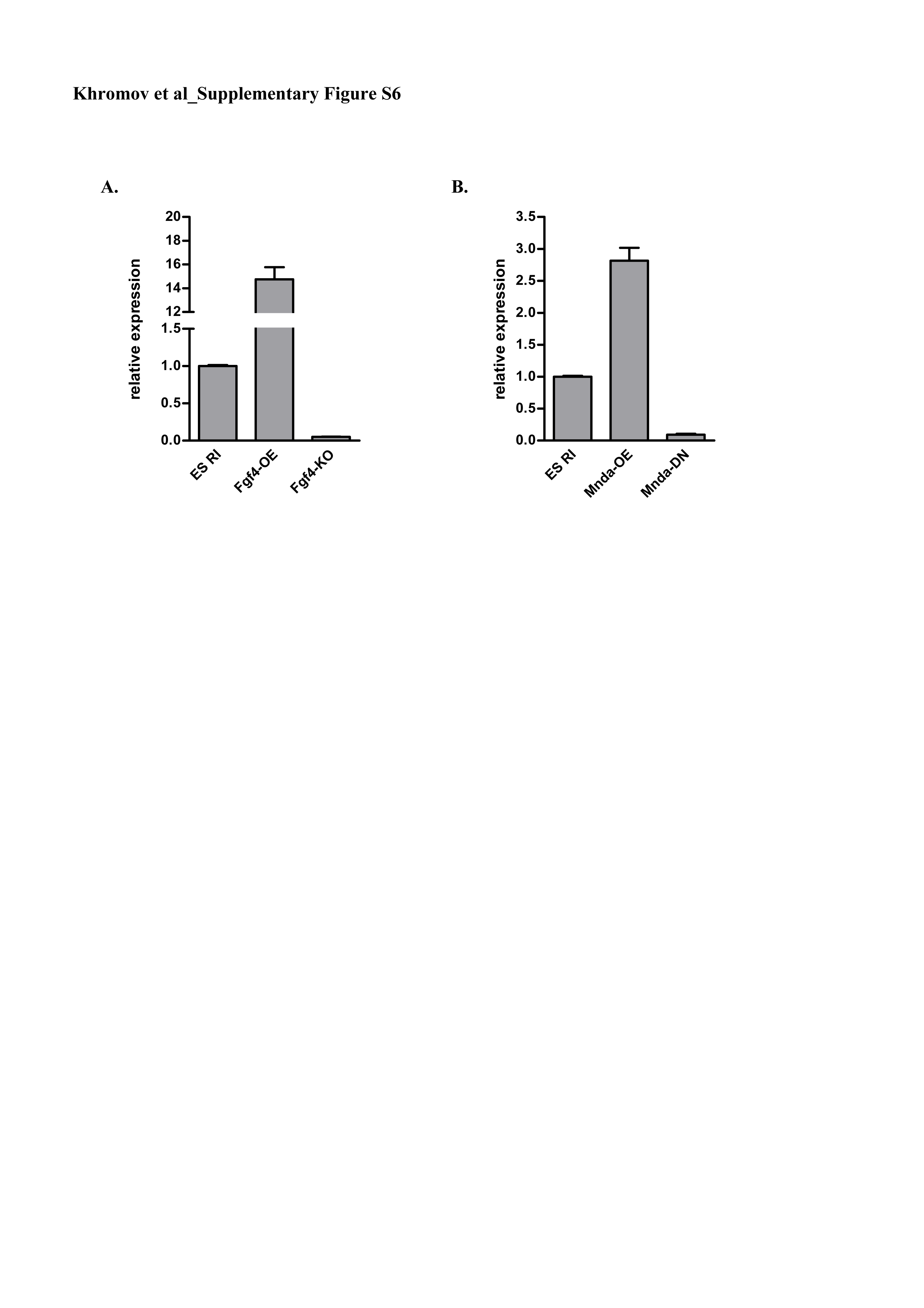

Supplement: Figure S6 — Expression analysis of overexpressing, knockdown, and knockout cell lines. (A) Real-time qPCR analysis showing the expression of Fgf4 in control, Fgf4-OE, and Fgf4-KO cells. (B) Real-time qPCR analysis showing the expression of Mnda in control, Mnda-OE, and Mnda-DN cells. (TIF) [file pone.0048869.s006.tif]

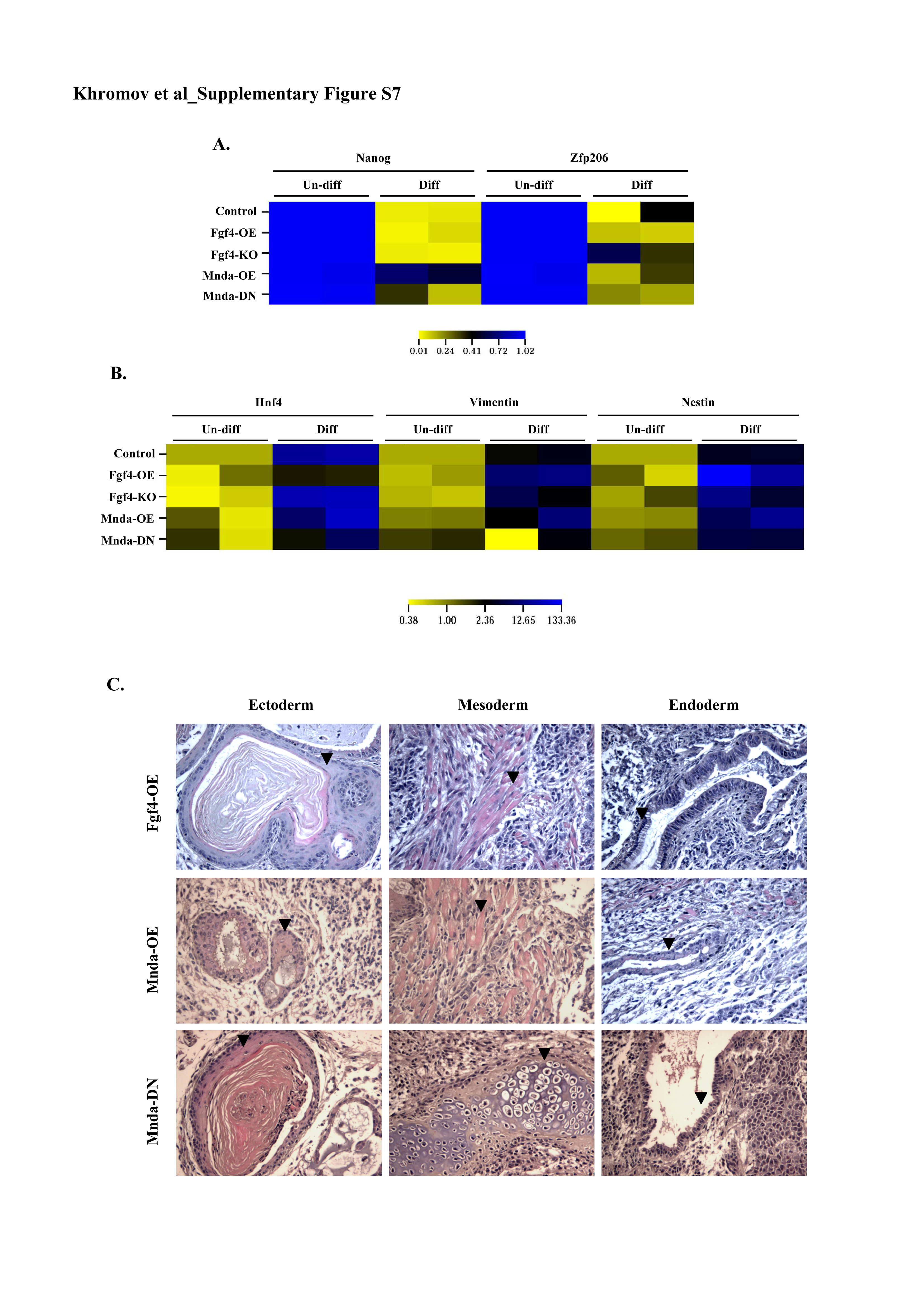

Supplement: Figure S7 — Differentiation potential of overexpression and knockdown cell lines. (A) Heatmap representing the qPCR data of pluripotency marker genes, Nanog and Zfp206 expression in undifferentiated and differentiated control ESCs, Fgf4-OE, Fgf4-KO, Mnda-OE, and Mnda-DN cell lines. The expression of Nanog and Zfp206 was reduced or absent in differentiated cells as expected. (B) Heatmap representing the qPCR data of differentiation marker genes, Hnf4, Vimentin, and Nestin expression in undifferentiated and differentiated control ESCs, Fgf4-OE, Fgf4-KO, Mnda-OE, and Mnda-DN cell lines. Expression of Hnf4, Vimentin, and Nestin was upregulated, indicating differentiation. (C) Tumors obtained from immunodeficient mice after injection of Fgf4-OE, Mnda-OE, and Mnda-DN ESCs were HE stained and identified as teratomas. The representative cell or tissue types for all three germ layers were indicated with arrowheads. (TIF) [file pone.0048869.s007.tif]

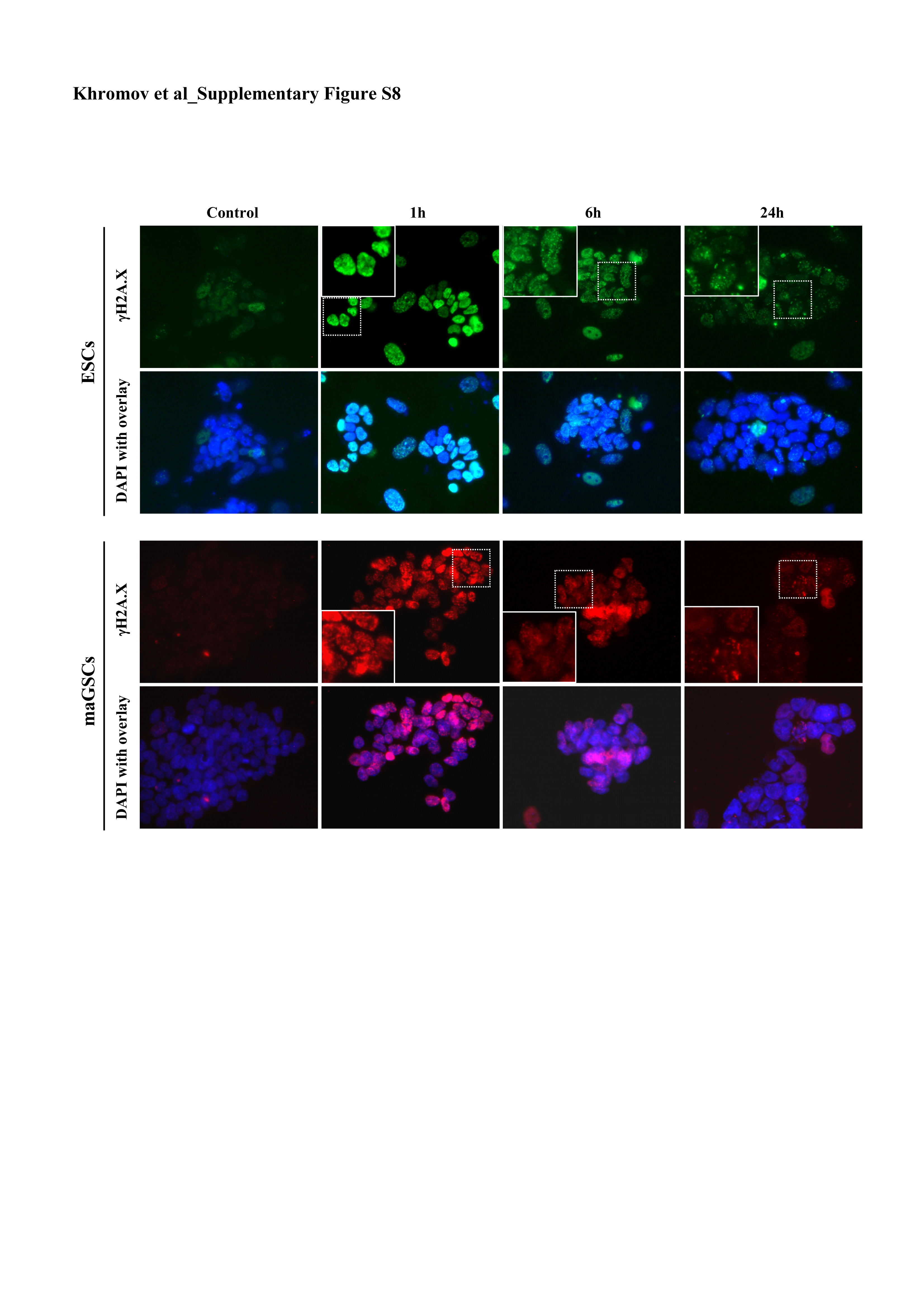

Supplement: Figure S8 — Analysis of NCS induced DNA damage in ESCs and maGSC. Wild-type ESCs (A) or maGSCs (B) were treated with NCS for 30 min and allowed to recover for 24 h. Immunostaining at indicated time points using γH2A.X (green) indicated the strong induction of DSBs by 1 h and gradual disappearance by 24 h, indicating DNA repair. Inset showing the γH2A.X foci of the enlarged region (dotted line). DAPI (blue) was used to stain the nucleus. (TIF) [file pone.0048869.s008.tif]

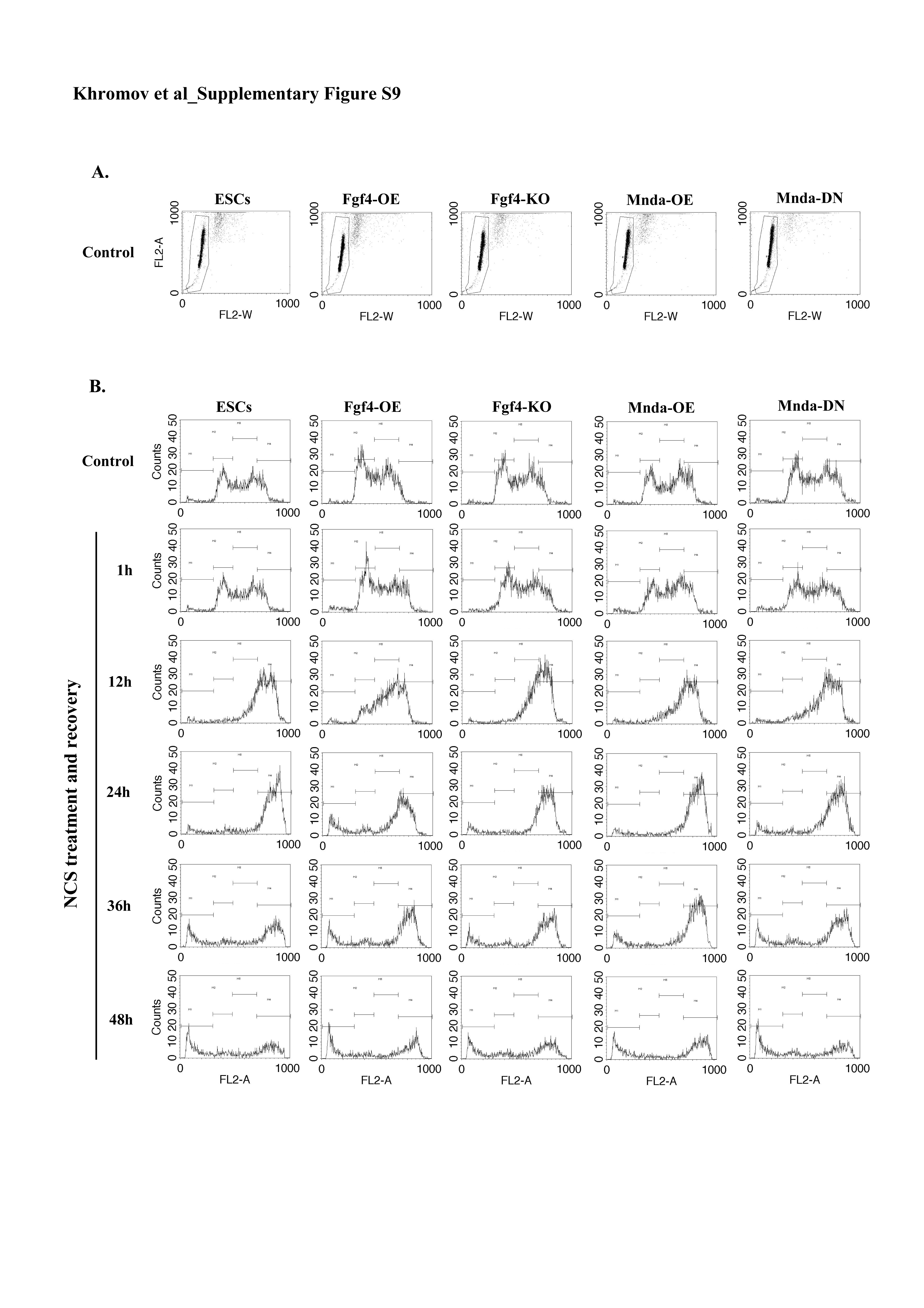

Supplement: Figure S9 — Cell cycle analysis parameters and patterns of DNA damage induced cells. (A) Representative images showing the gating parameters used to exclude doublets from the analysis. (B) Representative images showing the cell cycle patterns in control or NCS treated and recovered cells at indicated time points. (TIF) [file pone.0048869.s009.tif]
