# Supplementary material for: Apoptosis-Related Gene Expression Profiles of Mouse ESCs and maGSCs: Role of Fgf4 and Mnda in Pluripotent Cell Responses to Genotoxicity
Source: PLoS One. 2012 Nov 7;7(11):e48869. doi: 10.1371/journal.pone.0048869 (PMC3492253; doi:10.1371/journal.pone.0048869)
Supplement: Table S1 — Primers used in this study. (DOC) [file pone.0048869.s010.doc]

**Supplementary Table S1. Primers used in** this study

| **Gene** | **Forward primer sequence** | **Reverse primer sequence** |
| --- | --- | --- |
| *Rpl13* | 5’-GCTCCAAGCTCATCCTGTTC-3’ | 5’-CTCTGGCCTTTTCCTTTTTG-3’ |
| *Hprt* | 5'-CGTCGTGATTAGCGATGATG-3’ | 5'-TATGTCCCCCGTTGACTGAT-3’ |
| *Fgf4* | 5’-GTGGTGAGCATCTTCGGAGT-3’ | 5’-GTACGCGTAGGCTTCGTAGG-3’ |
| *Nanog* | 5’-TTACAAGGG TCTGCTACTGAGATG-3’ | 5’-CAGGACTTGAGAGCTTTTGTTTG-3’ |
| *Zfp206* | 5’-GAGAGGAGGTGGTACAGCTATTG-3’ | 5’- AGGTGGAGGTAACTCATTCAGTG-3’ |
| *Hnf4* | 5’-CCACATGTACTCCTGCAGGTTTAG-3’ | 5’-CGCTCATTTTGGACAGCTTC-3’ |
| *Nestin* | 5’-CTGCAGGCCACTCAAAACTT-3’ | 5’-ATTAGGCAAGGGGGAAGAGA-3’ |
| *Vimentin* | 5’-TGCAGTCATTCAGACAGGATGT-3’ | 5’-ATCTCTTCATCGTGCAGTTTCTTC-3’ |
| *Mmp10* | 5’-TGGATAAAGGCTTCCCAAGA-3’ | 5’-GGGGTCAAACTCGAACTGTG-3’ |
| *Atf5* | 5’-TATGAGGTCCTTGGGGGTG-3’ | 5’-ACCCGCTCAGTCATCCAAT-3’ |
| *Bok* | 5’-AGTCGGAGCCTGTGGTGAC-3’ | 5’-CCACGGAATACAGGGACACT-3’ |
| *P53* | 5’-CTAGCATTCAGGCCCTCATC-3’ | 5’-TCCGACTGTGACTCCTCCAT-3’ |
| *Bax* | 5’-TGTTTGCTGATGGCAACTTC-3’ | 5’-GATGGTTCTGATCAGCTCGG-3’ |
| *Nfkb1* | 5’-TTTCGATTCCGCTATGTGTG-3’ | 5’-GAACGATAACCTTTGCAGGC-3’ |
| *Hspa1a* | 5’-TTTGTGTTTGGACTCTCCCC-3’ | 5’-GCAAGGAGAAGCAGCAGAGT-3’ |
| *Hist2h2be* | 5’-AGGACTTCAAGACGGACCTG-3’ | 5’-AGGTTGGTGTCCTCGAACAG-3’ |
| *Mia2* | 5’-GGTACAAAGTTGCTGGCACA-3’ | 5’-CGGGTCCTGTGTAATCTCTCA-3’ |
